# Generation of Non-Small Cell Lung Cancer Patient-Derived Xenografts to Study Intratumor Heterogeneity

**DOI:** 10.3390/cancers13102446

**Published:** 2021-05-18

**Authors:** Zoi Kanaki, Alexandra Voutsina, Athina Markou, Ioannis S. Pateras, Konstantinos Potaris, Margaritis Avgeris, Periklis Makrythanasis, Emmanouil I. Athanasiadis, Ioannis Vamvakaris, Eleni Patsea, Konstantinos Vachlas, Evi Lianidou, Vassilis Georgoulias, Athanasios Kotsakis, Apostolos Klinakis

**Affiliations:** 1 Center for Basic Research, Biomedical Research Foundation of the Academy of Athens, 11527 Athens, Greece; zkanaki@bioacademy.gr (Z.K.); avoutsina@bioacademy.gr (A.V.); pmakrythanasis@bioacademy.gr (P.M.); 2 Analysis of Circulating Tumor Cells Lab, Lab of Analytical Chemistry, Department of Chemistry, National and Kapodistrian University of Athens, 15771 Athens, Greece; atmarkou@chem.uoa.gr (A.M.); lianidou@chem.uoa.gr (E.L.); 3 Department of Histology and Embryology, School of Medicine, National and Kapodistrian University of Athens, 11527 Athens, Greece; ipateras@med.uoa.gr; 4 Department of Thoracic Surgery, Sotiria Hospital for Chest Diseases, 11527 Athens, Greece; potaris.k@sotiria.gr (K.P.); lycomides@med.uoa.gr (K.V.); 5 Laboratory of Clinical Biochemistry–Molecular Diagnostics, Second Department of Pediatrics, School of Medicine, National and Kapodistrian University of Athens, “P. & A. Kyriakou” Children’s Hospital, 11527 Athens, Greece; mavgeris@med.uoa.gr; 6 Greek Genome Center, Biomedical Research Foundation of the Academy of Athens, 11527 Athens, Greece; mathan@bioacademy.gr; 7 Pathology Department, Athens Chest Hospital “Sotiria”, 11527 Athens, Greece; pathanman@sotiria.gr; 8 Department of Pathology, Metropolitan Hospital, 18547 Cholargos, Greece; pathologygeneral@metropolitan-hospital.gr; 9 Hellenic Oncology Research Group (HORG), 11471 Athens, Greece; eoeo@horg.gr; 10 Department of Medical Oncology, General University Hospital of Larissa, 41110 Larissa, Greece; thankotsakis@uth.gr

**Keywords:** non-small cell lung cancer, patient-derived xenografts, intratumor heterogeneity, genetic profiling

## Abstract

**Simple Summary:**

It is widely thought that tumors are composed of different subpopulations of cancer cells carrying genetic alterations with some of them being common among all cells while others are unique for each subpopulation. This variable genetic profile of tumor cells is a component of what is collectively described as intratumor heterogeneity (ITH). Surviving the immune system and therapies, and establishing metastases are forces of natural selection that act upon ITH and drive tumor evolution and, eventually, the clinical presentation of patients. The aim of this prospective study was to investigate ITH in early-stage operable non-small cell lung cancer. We directly grafted human tumors in immunosuppressed mice and compared the genetic profile of the tumors grown in mice with that of the original human tumors. We identified clinical factors that affected the ability of human tumors to grow as mouse xenografts.

**Abstract:**

Recent advances in sequencing technologies have allowed the in-depth molecular study of tumors, even at the single cell level. Sequencing efforts have uncovered a previously unappreciated heterogeneity among tumor cells, which has been postulated to be the driving force of tumor evolution and to facilitate recurrence, metastasis, and drug resistance. In the current study, focused on early-stage operable non-small cell lung cancer, we used tumor growth in patient-derived xenograft (PDX) models in mice as a fast-forward tumor evolution process to investigate the molecular characteristics of tumor cells that grow in mice, as well as the parameters that affect the grafting efficiency. We found that squamous cell carcinomas grafted significantly more efficiently compared with adenocarcinomas. Advanced stage, patient age and primary tumor size were positively correlated with grafting. Additionally, we isolated and characterized circulating tumor cells (CTC) from patients’ peripheral blood and found that the presence of CTCs expressing epithelial-to-mesenchymal (EMT) markers correlated with the grafting potential. Interestingly, exome sequencing of the PDX tumor identified genetic alterations in DNA repair and genome integrity genes that were under-represented in the human primary counterpart. In conclusion, through the generation of a PDX biobank of NSCLC, we identified the clinical and molecular properties of tumors that affected growth in mice.

## 1. Introduction

Lung cancer is by far the deadliest type of cancer [1] with the majority of cases corresponding to non-small cell lung cancer (NSCLC). The 5-year survival rate of patients with metastatic disease is dismal (6%), while patients with loco-regional disease have a better prognosis depending on the Tumor Node Metastasis (TNM) stage (cancer.gov). While molecular profiling has allowed the identification of several mutations that can be targeted with novel drugs, it has also uncovered extensive intratumor heterogeneity (ITH) [2]. ITH is a common feature of many cancers and has been implicated in disease recurrence and therapeutic response [3].

Mouse models have historically facilitated the study of tumor biology and have been used for the preclinical testing of novel drugs. More recently, the development of patient-derived xenograft (PDX) models has allowed investigators to generate more faithful models of the human disease by engrafting primary tumor specimens directly into immunocompromised mice [4,5]. The reported grafting efficiency varies among studies and tumor types, with melanoma and head and neck carcinoma showing the highest grafting rates while renal cell carcinoma and breast tumors were found at the other end of the scale [6]. 

In addition to tumor type, other factors, such as the degree of mouse immunodeficiency (i.e., which immunocompromised strain is used) and size of the grafted specimen could also be important factors. Regarding NSCLC, PDXs have been extensively used in preclinical studies targeting mostly EGFR [4,7,8]. Interestingly, published studies have reported a higher grafting efficiency for lung squamous cell carcinomas (LUSC) in comparison to lung adenocarcinomas (LUAD) [9,10]. With the exception of concurrent distant metastases and advanced disease stage [11], most studies fail to identify clinicopathological characteristics, such as age, smoking, or genetic profile, to be associated with the in vivo take rate. 

A common observation is the high histological resemblance between the primary tumor and early passage mouse xenografts [4,7,9,12,13]. More recent reports have focused on the comparison of the molecular characteristics of primary tumors and the respective mouse counterparts. While some clonal evolution is identified in the PDX tumors, as indicated by the increased frequency of primary tumor genetic alterations [12,13,14], we are far from understanding how certain subclones within the primary tumor are favored in mice.

The purpose of the current study was to assess how ITH within the primary tumor impacts the grafting efficiency in early-stage NSCLC and how the grafting efficiency correlates with the tumor stage, circulating tumor cell (CTC) count, disease recurrence, progression, and ultimately patient outcome. A consortium of surgeons, oncologists, and pathologists together with scientists in the fields of clinical chemistry, molecular diagnostics, and mouse genetics came together and generated a large panel of fully characterized NSCLC PDXs. Here, we present this panel of PDX models and discuss the molecular and clinical aspects of both the primary and PDX tumors.

## 2. Materials and Methods

### 2.1. Patient Recruitment

Eighty-two patients with a median age of 65.5 years (range, 39–86) with histologically documented NSCLC and operable disease were enrolled in the study. All patients gave their written informed consent to participate in the study, which was approved by the Ethics and Scientific Committee of the Metropolitan General hospital (308/28-12-2017). All patients were operated in the “Sotiria” General Hospital (Athens) by the same surgery team (K.P. and K.V.). There were 52 male and 30 female, and 44 (53.7%) of them had an adenocarcinoma (LUAD) histology. The pathological staging revealed that 39 patients had stage I (A and B), 25 had stage II (A and B) and 18 had stage IIIA tumors. Fifty-five patients (67.1%) had no evidence of disease dissemination in resected lymph nodes (N0 disease).

### 2.2. PDX Generation

All animal experiments were performed according to national and international regulations and were approved by the BRFAA (Biomedical Research Foundation of the Academy of Athens, Athens Greece) ethical committee. NOD.Cg-Prkdc^scid^ Il2rg^tm1Wjl^/SzJ (Stock No: 005557; NSG) [15,16] mice were purchased from the Jax repository (Bar Harbor, ME USA) and bred in-house. All xenotransplantations were performed within 6 h from the patient’s surgery due to the close proximity of the hospital and the mouse facility. Tumor specimens up to 5 mm (depending on tissue availability) were transplanted subcutaneously in NSG mice under anesthesia [17]. All fragments were mirror images of tumor segments, which were submitted for molecular analysis by two independent histologists. 

Prior to engraftment, a small piece of the tissue was snap frozen and was later processed for molecular analysis. Mice were kept in pathogen-free conditions throughout their life. All tumors were passaged at least once when they reached a maximum diameter of 1 cm. The growth time from implantation to passage 1 varied from 1 to 4 months. Grafted tumors that failed to grow within 4 months were considered “no-take”. During passage 1, a portion of the PDX tumor was processed for molecular and histological analysis [18] by two pathologists. No distant metastases were ever observed. All early passages were also live-frozen in order to generate a biobank of early passage NSCLC PDXs.

### 2.3. Histopathological Examination

For the histopathological assessment, we performed hematoxylin (Millipore, Burlington, MA, USA) and eosin (DIAPATH, Martinengo, BG, Italy) (H/E) staining along with immunohistochemical analysis employing anti-TTF1 (SPT24, 1:100 dilution, Leica Biosystems Newcastle Ltd. Balliol Business Park, Benton Ln, Newcastle upon Tyne, United Kingdom) and anti-P40 (BC28, ready to use, Roche, Basel, Switzerland).

### 2.4. DNA/RNA Extraction

The cellularity of the primary tumor was determined in FFPE tissue using H/E stained sections. Sections (four to five sections at 10 μm each) with a minimum 20% tumor content were selected and used for DNA isolation using the Qiagen FFPE DNA isolation kit (QIAGEN, Hilden, Germany) as per the manufacturer’s instructions and quantified using the Qubit fluorometer using the Qubit BR DNA assay kit (Thermo Fisher Scientific, Waltham, MA, USA). 

### 2.5. Library Preparation and Sequencing

Custom capture probes were designed using SureDesign (Agilent Technologies, Santa Clara, CA, USA) targeting all exons in 58 genes (Table S1). A total of 50–100 ng of DNA from FFPE tissue samples were used for library preparation and sequencing. The library preparation for each sample was done using the SureSelect XT HS Reagent Kit (Agilent Technologies, Santa Clara, CA, USA) according to the manufacturer’s instructions. In brief, pre-enriched adapter-ligated libraries were prepared, and custom capture probes were hybridized to target sequences to allow for sequence enrichment using streptavidin beads. The post-enriched libraries were quantified using the Qubit dsDNA Assay Kit (Thermo Fisher Scientific, Waltham, MA, USA), and the library quality was assessed using the Agilent 2100 bioanalyzer (Agilent Technologies, Santa Clara, CA, USA). The libraries were pooled to equimolar concentrations and sequenced on a NextSeq 500 (Illumina, San Diego, CA, USA).

The NGS panel was developed to cover ~289,255 kbp genomic regions of 58 genes, including the majority of known oncogenes and tumor suppressors in NSCLC and several genes involved in DNA damage response/DNA repair mutations, which could affect the therapeutic decisions. All sequencing was paired-end with read lengths of 150 bp. The average total number of reads across the specimen data sets was 29.8 million. The coverage across the capture regions was assessed using mosdepth [19]. The percentage of base positions at which on-target reads were achieved at depths of 100×, 500×, and 1000× was calculated. For an average specimen, 96.4% of the positions were covered at a depth of 100×, 88.9% at a depth of 500×, and 76.8% at a depth of 1000×.

Sequencing data analysis was performed using bwa 0.7.17-r1188 [20], samtools 1.9 [21], GATK 4.1.2 [22], and ANNOVAR 2018-04-16 [23] in a sequential manner. As cutoff for the analysis, 5% variant allele frequency (VAF) was used and at least 100x depth. Artifacts and common variants were filtered out based on frequency detection in our sample (10%). Differences in the VAF and unique variants in different libraries originating from samples from the patient were identified by direct comparison of the variants’ list.

### 2.6. Primary and PDX Tumor Comparison

For identification of primary and PDX tumor genetic alterations, the cut-off allele frequency was set at 0.5% for the primary tumor and 5% for the mouse counterpart. Therefore, it was possible to identify genetic alterations that were represented in a large portion of the PDX even if they started from a very low representation in the primary tumor. To exclude passenger mutations in the specific tumor specimen that were coincidentally enriched during the development of the PDX, other genetic alterations in the same primary tumor-PDX pair were used as normalizers. The human tumor cellularity was taken into account, and samples with cellularity < 50 were excluded from the comparison.

### 2.7. CTC Enrichment for Molecular Analysis

Peripheral blood PB (25 mL) was collected prior to surgery in EDTA tubes after discarding the first 5 mL of blood draw to avoid contamination of skin epithelial cells. CTC enrichment was performed using the size-based microfluidic device Parsortix (ANGLE plc, Guildford, Surrey, UK), which contains a microscope slide sized disposable cassette [24,25]. CTCs were finally harvested in a total volume of 200 μL of PBS, and the total RNA was extracted from the harvested cells using TRIZOL-LS (ThermoFisher Scientific, Waltham, MA, USA). Finally, cDNA synthesis was performed as previously described [26].

### 2.8. Gene Expression Analysis of CTCs by RT-qPCR 

RT-qPCR was performed to evaluate the gene expression of epithelial (CK-8, CK-18, and CK-19), and mesenchymal/EMT (VIM and TWIST1) markers. B2M was used as a reference gene for relative quantification but also for ensuring the presence of amplifiable material in all samples and to avoid false-negative results [27], and RT-qPCR was performed as previously reported [28].

In all patient samples, RT-qPCR data for *VIM* were normalized in respect to the expression of *B2M* reference gene by using the 2^–ΔΔCq^ approach. The cut-off value for *VIM* transcripts was calculated as the mean of signals derived in the healthy donor group plus 2SD. Using this approach, a sample was considered to overexpress *VIM* based on the fold change of *VIM* expression in the CTC fraction with respect to the corresponding “CTC” fraction in the group of these healthy individuals.

### 2.9. Statistical Analysis

Statistical analysis was performed using IBM SPSS Statistics 20 software (IBM Corp., Armonk, New York, USA). The association of PDX groups (PDX+ and PDX–) with categorical variables was evaluated by Fisher’s Exact and Pearson chi-square tests, while the correlation with continuous data was assessed with the non-parametric Mann–Whitney U test for variables with two categories and Kruskal–Wallis tests for variables with more than two categories. Patient death and treatment failure were assessed as clinical end-point events for the survival analysis by Kaplan–Meier curves using the log-rank test.

## 3. Results

### 3.1. Establishment of NSCLC PDXs

Following surgery, sufficient material for grafting in at least one NSG mouse was available from 52 patients. The complete clinical and pathological patient characteristics are provided in Table 1.

**Figure 1:**
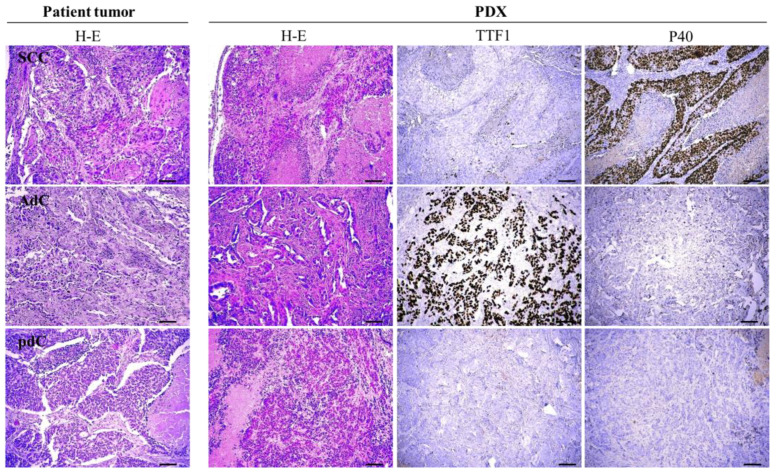
Histological evaluation of primary tumors and PDX. Images from eosin and hematoxylin staining (H/E) from a representative example of squamous cell carcinoma (SCC), adenocarcinoma (AdC), and a poorly differentiated carcinoma (pdC) showing that the mouse tumor preserved the morphological features of the primary tumor. The squamous cell marker P40 and the adenocarcinoma marker TTF1 were also used on the PDX tumor. Scale bar: 100 μm.

Thirty-one (59.6%) of the 52 specimens yielded successful xenografts, which were passaged at least once before freezing. During the first passage, part of the PDX tumor was fixed in formalin and processed with H/E staining for histopathological characterization in comparison with the corresponding patients’ primary tumors. Microscopical examination revealed that PDXs preserved the histopathological characteristics of the original tumors (Figure 1). 

The phenotype of squamous cell carcinoma and adenocarcinoma in the primary tumors was faithfully retained in PDX, verified by the staining pattern of the lung adenocarcinoma (LUAD) marker TTF1 and the lung squamous cell carcinoma (LUSC) marker p40 (Figure 1). Figure 1 also depicts a case of a poorly differentiated primary lesion that fully retained the same differentiation pattern in the mouse.

### 3.2. Clinical Characteristics and Grafting Efficiency

We performed statistical analysis to identify clinical parameters that could impact the grafting efficiency. All *p* values are provided in Table 1. Although it did not reach statistical significance, the grafting efficiency was higher among men and current smokers who, as expected, represented the majority of cases. In agreement with reported data [10], adenocarcinomas grafted poorly (*p* = 0.048) in comparison with the other tumor types (essentially LUSC). According to our experience regarding the generation of PDXs from head and neck squamous cell carcinoma, squamous cell carcinomas, in general, graft with a high efficiency (unpublished data).

The grafting efficiency positively correlated with the tumor/clinical stage, with 6/6 T4 and 12/24 stage IIIA tumors leading to successful grafting in mice, and with advanced age (*p* = 0.03; Table 1), possibly reflecting late diagnosis and a higher tumor burden. Indeed, the median size of primary tumors with successful grafting in mice was 33.7 cm^3^ in comparison to the 14.7 cm^3^ of those that failed to grow in mice (*p* = 0.07; Table 1). Interestingly, we did not observe any correlation with the disease-free survival or overall survival (Figure S1A,B).

### 3.3. Correlation between the Presence of CTCs and Grafting

Patient CTCs were detected and characterized based on the expression of epithelial (*KRT8*, *KRT18,* and *KRT19*) and mesenchymal (*Vim* and *Twist1*) markers. The gene expression analysis of the above markers demonstrated significant heterogeneity among NSCLC patients. CTCs were detected in 47 out of 52 (90.4%) patients. At least one epithelial marker was detected in 19 out of 52 (36.5%) patients; *KRT8* transcripts were detected in 8 out of 47 (17.0%), *KRT18* transcripts were detected in 7 out of 47 (14.9%), and *KRT19* transcripts were detected in 12 out of 47 (25.5%) samples before surgery (Table 2).

Similarly, at least one mesenchymal/EMT marker, was detected in 34 out of 47 (72.3%) patients; *VIM* transcripts were detected in 27 out of 47 (57.4%) patients, whereas *TWIST1* transcripts were detected in 12 (25.5%) patients (Table 3).

The grafting efficiency showed a not statistically significant positive trend with CTCs expressing EMT markers *TWIST* (*p* = 0.179) and *VIM* (*p* = 0.231). The expression of either showed an even stronger trend (*p* = 0.107) (Table 3). Interestingly, only *VIM* was detected in healthy donors (HD), the peripheral blood of which was subjected to the same process. Thus, we were able to compare *VIM* expression in the HD group against surgical samples that did or did not yield PDXs. As Figure S2 indicates, *VIM*-overexpressing CTCs were found in patients that also grafted successfully.

There was no correlation between the grafting efficiency and the expression of epithelial markers. However, it should be noted that the KRT18 expression, which mainly characterizes LUAD, absolutely correlated with the grafting (7/7 cases, *p* = 0.009) (Table 3), corresponding to two LUAD, four LUSC, and one large cell carcinoma case. KRT19, on the other hand, which is expressed by both LUAD and LUSC, did not show any correlation with the grafting efficiency [29], despite the fact that it has been shown to be an excellent marker for the detection of lymph node metastases of NSCLC [30].

### 3.4. Molecular Comparison of PDX and Primary Tumors

Given the well documented ITH, we aimed to investigate whether certain genetic alterations in primary tumor subclones might confer an advantage for efficient grafting in mice. To this direction, we used our custom panel for targeted exome sequencing and generated the genetic alteration profile of the primary tumor and the mouse counterpart. A summary of pathogenic genetic alterations or mutations with unknown clinical significance identified in primary tumors which successfully grafted in mice is provided in Table S2. Assuming that certain subclones are favored during tumor growth in mice, this would lead to an enrichment of certain genetic alterations present only within a subset of tumor cells. 

This enrichment would be reflected in the VAF, which would be expected to be higher in the PDX tumor in comparison to the primary one. To identify such cases, the genetic profile of the mouse tumors from passage 1 of the PDXs was compared with the genetic profile of the human counterpart. Interestingly, the most striking examples of mutations that were underrepresented in the primary tumor but were subsequently enriched in the mouse counterpart were found in DNA repair and genome integrity proteins, with the most prominent being TP53 (Table 4).

## 4. Discussion

A personalized approach in cancer therapeutics has long been a noble goal for both clinicians and patients. More sophisticated therapies, often targeting specific mutations are very helpful in this direction. On the other hand, clinical practice has invariably proven that tumor cells develop resistance to targeted therapies [31]. To this point, it is not clear to what extent this therapeutic resistance is intrinsic due to a minority of cells within the tumor that outcompete their less resistant counterparts or whether resistance is an acquired property [31]. 

Even in the cases of acquired resistance, it is safe to assume that the genetic plasticity of cancer cells and novel acquired genetic alterations help them bypass the toxicity induced by therapeutic agents. On the other hand, tumors with high genetic heterogeneity are much more likely to develop resistance because the combination of genetic events leading to this resistance is easier to achieve. Recent studies have indicated that, even for a single targeted therapy (e.g., EGFR inhibition in NSCLC) a multitude of resistance mechanisms have been described [32,33,34].

Patient-derived xenografts have proven their usefulness in studying various aspects of tumor biology. Not only do they recapitulate the human disease, but they also are almost unlimited sources of biological material. They are particularly useful in preclinical studies for testing new therapeutic regimens [35,36]. In this regard, it is worth mentioning that we have obtained PDXs from tumors carrying EGFR mutations in exons 19 and 20 (Table S2). Patients with EGFR mutations in exons 17–21 have a heterogeneous response to EGFR tyrosine kinase inhibitors as indicated by clinical and experimental data in patients, mouse models, and cell lines [37]. Models, such as the ones described here, could become useful tools in understanding the underlying mechanism for this variable response and in identifying additional biomarkers for clinical use. Moreover, we obtained 4 LUAD PDXs with KRAS mutations (Table S2). In agreement with previous reports [38], KRAS^G12C^ is the most common KRAS variant and is currently targetable with clinically-approved covalently-bound inhibitors [39,40]. While clinical trials with various KRAS^G12C^ inhibitors are in progress, data from patients and mouse models point to heterogeneous responses and therapeutic resistance as emerging issues [41]. Again, preclinical models, such as those described here, combined with clinical data, will be invaluable tools in understanding the mechanistic aspects of these issues but also to better stratify patients and possibly design improved inhibitors and therapeutic schemes.

Similarly to the metastatic potential and the therapeutic response in humans, the factors affecting the grafting efficiency are largely unknown. In the current study, we asked the question of whether PDX can be useful in studying ITH. Considering that growth in mice imitates metastasis, PDXs provide a platform for fast forwarding tumor evolution, which favors certain tumor cells within a heterogeneous primary tumor. Therefore, a panel of PDXs was developed from patients with early-stage operable NSCLC who had been fully characterized both molecularly and histologically. 

The presented data seem to indicate that squamous cell carcinomas grafted more efficiently than adenocarcinomas, a property most likely inherent in squamous cell carcinomas in general. Additionally, the grafting efficiency positively correlated with the tumor and disease stage as well as the primary tumor size. Rather surprisingly, we observed no correlation with disease progression and patient survival; however, this could be attributed mainly to the small numbers of events of the studied cohort due to the patients’ clinical characteristics (mainly with stage I and II presenting a relatively low incidence of disease relapse, as well as the short duration of the follow-up period).

The isolation and characterization of CTCs indicated that CTCs expressing EMT markers were associated with PDX growth, in concordance with the long-known ability of tumor cells that have undergone EMT to escape the primary tumor site and form metastases [42]. Our analysis also revealed that PDX development in mice correlates with the expression of *KRT18* in CTCs. Earlier studies suggested a link between CK18 protein expression and unfavorable clinicopathological features and outcomes in squamous cell carcinomas [43]. To our knowledge, this is the first time that the CTC count and specific markers expressed by CTCs have been linked with the grafting efficiency in NSCLC. The biological underpinnings of these findings are of great interest and will fuel future studies.

As expected, primary tumor driver mutations were invariably found in the PDX counterparts. On the other hand, several genetic alterations, which were found at low frequencies in the human specimen, were significantly enriched in mice following successful grafting. The majority of those were in the *TP53* locus; however, genetic alterations were also found in the *CDKN2A*, *RB1*, and *BRCA2*, as well as the *STK11* locus (also known as *LKB1*), which is a well-known tumor suppressor in lung cancer [44]. 

We cannot exclude the possibility that, in certain cases, originally heterozygous mutations reach a variant allele frequency close to 1 due to loss-of-heterozygosity (LOH). LOH has been well documented in NSCLC for the *TP53* [45] and the *RB1* [46] loci. Moreover, it has been shown that missense single amino acid variations in the DNA binding domain often leads to gain-of-function TP53 proteins, which favor disease progression and metastasis [47,48]. These findings imply that, indeed, there is some kind of cancer cell selection for growth in mice, and that this selection process could favor cells with defects in genome integrity and cell cycle checkpoints. 

This could be an indication that high-speed tumor evolution fueled by defects in the DNA repair and genome surveillance machineries might be the driving force in mouse grafting. Interestingly, previous studies focusing on tumor evolution during PDX growth did not identified genetic alterations in the aforementioned genes being favored in mice [12,13,14]. On the other hand, the panel of genes assessed in this study was enriched in genes encoding proteins involved in DNA damage response and DNA repair as well as cell cycle regulation. 

This is one primary limitation in this study, i.e., assessing the mutation status of a rather short list of genes. Thus, more detailed molecular characterization in the form of whole exome sequencing of the described primary and PDX tumors would likely provide a more complete picture. In fact, whole exome coverage and the assessment of structural variants in the form of fusions and copy number variations (events that our approach cannot detect) would likely identify other gain- and loss-of-function genetic events favoring tumor growth in mice. Moreover, the single cell genomics of tumor cells and/or CTCs would also be valuable in such studies.

## 5. Conclusions

Herein, we presented the generation and characterization of a PDX panel from early-stage operable NSCLC. We found correlations between the tumor growth in mice and a number of clinicopathological characteristics, including the tumor grade, stage, and size. By analyzing CTCs, we uncovered a positive correlation between CTCs expressing EMT markers and the epithelial marker KRT18 and the ability of the respective primary tumor to grow in mice. More importantly, we identified a positive selection in mice of cancer cells/subclones carrying genetic alterations, mostly in TP53, but also in other genome surveillance and cell cycle control proteins.

## Figures and Tables

**Table 1:** Clinical and pathological characteristics of the patients.

Patient Characteristics	PDX YES (*n* = 31)	PDX NO (*n* = 21)	*p*-Value
Categorical Variables	*n* (%)	*n* (%)	
*Gender*			0.141 ^a^
Male	23 (74.2%)	11 (52.4%)	
Female	8 (25.8%)	10 (47.6%)	
*Age*			0.090 ^a^
<68y ^b^	10 (34.5%)	12 (60.0%)	
≥68y	19 (65.5%)	8 (40.0%)	
*Smoking status*			0.497 ^c^
Current	19 (61.3%)	10 (47.6%)	
Previous	6 (19.4%)	7 (33.3%)	
Never	6 (19.4%)	4 (19.0%)	
*Histological type*			0.100 ^c^
Adenocarcinoma	10 (32.3%)	13 (61.9%)	
Squamous	17 (54.8%)	7 (33.3%)	
Large cell or Sarcomatous	4 (12.9%)	1 (4.8%)	
*Clinical stage*			0.040 ^c^
I	13 (41.9%)	10 (47.6%)	
II	6 (19.4%)	9 (42.9%)	
IIIA	12 (38.7%)	2 (9.5%)	
*Tumor stage*			0.038 ^c^
pT1	5 (16.1%)	9 (42.9%)	
pT2	15 (48.4%)	7 (33.3%)	
pT3	5 (16.1%)	5 (23.8%)	
pT4	6 (19.4%)	0 (0%)	
**Continuous variables**	**Median (min–max)**	**Median (min–max)**	
*Tumor volume (cm^3^)*			0.070 ^d^
	33.7 (0.48–572.0)	14.7 (2.14–245.0)	
*Age (years)*			0.030 ^d^
	71 (56–81)	64 (40–75)	

^a^ Fisher’s Exact test; ^b^ Median age of patients with tumor used in xenotransplantations; ^c^ Pearson chi-square; and ^d^ Mann–Whitney U test.

**Table 2:** CTCs expressing epithelial markers and the grafting efficiency.

		PDX		Total	*p* Value ^a^
		No (−)	Yes (+)		
*KRT18*	Neg	21	19	37	*p* = 0.009
	Pos	0	7	7	
Total		21	26	47	
*KRT19*	Neg	16	19	34	*p* = 1.000
	Pos	5	7	12	
Total		21	26	47	
*KRT8*	Neg	17	22	39	*p* = 1.000
	Pos	4	4	8	
Total		21	26	47	
Epithelial markers	Neg	14	14	28	*p* = 0.377
	Pos	7	12	19	
Total		21	26	47	

^a^ Fisher’s exact test.

**Table 3:** CTCs expressing EMT markers and the grafting efficiency.

		PDX		Total	*p* Value ^a^
		No (−)	Yes (+)		
*VIM*	Neg	11	9	20	*p* = 0.231
	Pos	10	17	27	
Total		21	26	47	
*TWIST1*	Neg	18	17	35	*p* = 0.179
	Pos	3	9	12	
Total		21	26	47	
EMT markers (*VIM* & *TWIST1*)	Neg	8	5	13	*p* = 0.107
	Pos	13	21	34	
Total		21	26	47	

^a^ Fisher’s exact test.

**Table 4:** Molecular differences between primary tumors and PDX.

CODE	TYPE	GENETIC ALTERATIONS	TUMOR CELLULARITY (%)	VAF^PRIM.TUMOR^	VAF^PDX^
**105**	LUSC	**TP53:c.469G>T:p.V157F** KMT2C:c.918T>G:p.Y306*	90	**0.057** 0.059	**1** 0.062
**110**	LUSC	**TP53:c.413C>T:p.A138V** ALK:c.2712T>A:p.H904Q	85	**0.432** 0.36	**0.952** 0.191
**112**	PLEIO/LUAD	CTNNB1:c.98C>T:p.S33F TP53:c.811G>T:p.E271* STK11:c.487G>T:p.G163C KDR:c.2761delinsAT:p.F921Ifs*13 KDR:c.2757C>G:p.C919W	80	0.49 **0.436** 0.356 0.316 0.317	0.868 **0.93** 0.895 0.83 0.831
**302**	LUSC	KIT:2508G>T:p.M836I EGFR:c.1150A>T:p.T384S, MEN1:c.184A>G:p.T62A, MET:c.1241A>G:p.D414G	70	0.28 0.392 0.325 0.64	**0.973** **0.993** **0.644** 0.995
**512**	LUSC	**TP53:c.845G>C:p.R282P** CDKN2A:c.316_317T:p.V106Cfs*39 PDGFRA:c.1102G>C:p.E368Q	75	**0.336** **0.375** 0.4	**0.999** **0.998** 0.995
**517**	LUSC	RB1:c.852delinsTA:p.I285Nfs*2	75	**0.249**	**0.998**
**518**	LUSC	**TP53:c.659A>G:p.Y220C**	80	**0.304**	**0.996**
**519**	LCNEC	RB1:c.1953_1954T:p.V654Cfs*3	80	**0.732**	**0.998**
**531**	LUSC	**TP53:c.747G>T:p.R249S** CDKN2A:c.262G>T:p.E88* **PIK3CA:c.1633G>A:p.E545K**	80	**0.574** **0.416** **0.251**	**0.998** **0.991** **0.337**
**546**	LUAD	**KRAS:c.35G>A:p.G12D** ATM:c.8851G>A:p.V2951I	85	**0.342** 0.371	**0.296** **0.997**
**551**	LUSC	MTOR c.1333A>G:p.R445G CDKN2A:c.61G>C:p.A21P	65	0.686 0.295	0.994 **0.966**
**568**	LUAD	TP53:c.880G>T:p.E294* **KRAS:c.34G>T:p.G12C**	65	**0.09** **0.16**	**0.63** **0.62**
**569**	LUSC	**NF1:c.4662-1G>T** **TP53:c.994-2A>T;** SMAD4:c.1326G>T:p.Q442H	80	**0.301** **0.274** **0.145**	**0.984** **0.991** **0.953**
**571**	LUSC	CHEK2: c.1114C>T:p.Q372* TP53:c.994-2A>T BRCA2:c.3668A>G:p.H1223R NOTCH1:c.2058_2059T:p.C687Afs*84	75	0.294 **0.287** **0.59** **0.279**	0.362 **0.952** **0.959** **0.951**

Pathogenic events are shown in bold.

## Data Availability

The data presented in this study are available in Table 4 and Table S2.

## Supplementary Materials

The following are available online at https://www.mdpi.com/article/10.3390/cancers13102446/s1, Figure S1. Survival curves of patients operated with early-stage non-small cell lung cancer. Figure S2. *VIM* expression in healthy and NSCLC samples. Table S1: Panel of genes assessed with targeted exome sequencing; Table S2: Molecular profile of successfully grafted primary tumors.
