# Supplementary material for: Generation of Non-Small Cell Lung Cancer Patient-Derived Xenografts to Study Intratumor Heterogeneity
Source: Cancers (Basel). 2021 May 18;13(10):2446. doi: 10.3390/cancers13102446 (PMC8157865; doi:10.3390/cancers13102446)
Supplement: Supplementary file 1 [file cancers-13-02446-s001.zip › cancers-1192337-supplementary.pdf]

Supplementary materials

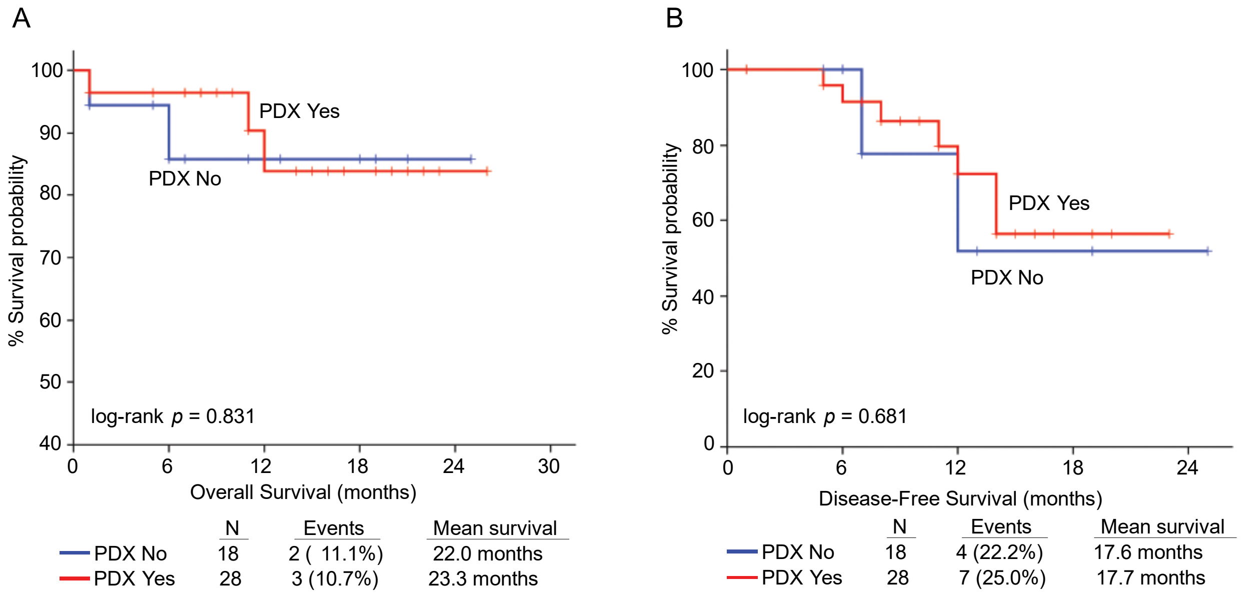

Figure S1. Survival curves of patients operated with early stage nonsmall cell lung cancer. Kaplan-Meier survival curves of patients indicating overall (**A**) and disease-free (B) survival of patients with respect to successful (PDX Yes) or unsuccessful (PDX No) of resected surgical material in mice.

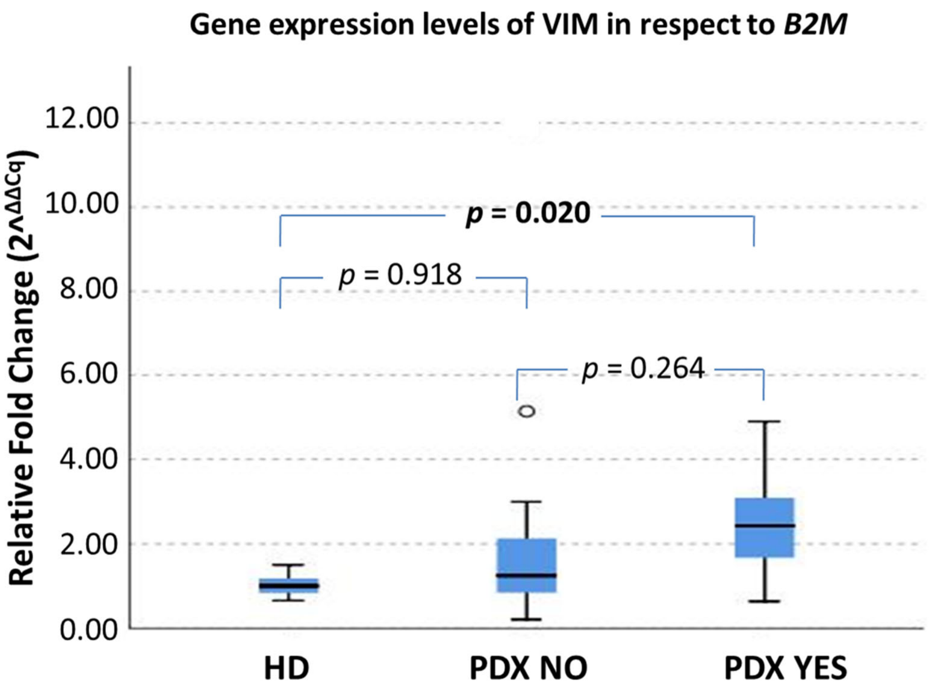

Figure S2. ***VIM*** **expression in healthy and NSCLC samples.** *VIM* expression levels for size-based CTC-enriched fractions from healthy donor (HD) and NSCLC patient with unsuccessful (PDX NO) and successful (PDX YES) grafting. Values are presented as relative fold change ($2^{-\Delta\Delta Cq}$) with respect to B2M expression. Kruskal-Wallis test was performed to statistical significance.

Table S1. Panel of genes assessed with targeted exome sequencing.

AKT1	*BRCA1*	*CDKN2A*	*ERBB4*	*IDH2*	*MLH1*	*KRAS*	*NOTCH1*	*PTEN*	*SMAD4*
ALK	*BRCA2*	*CHEK1*	*ESR1*	*JAK2*	*MAP2K1*	*MSH2*	*NRAS*	*RAD50*	*STAT3*
AR	*CCND1*	*CHEK2*	*FGFR1*	*JUN*	*MDM2*	*MSH6*	*PALB2*	*RAD51*	*STK11*
ATM	*CCNE1*	*CTNNB1*	*FGFR2*	*KDR*	*MDM4*	*mTOR*	*PDGFRA*	*RB1*	*TP53*
BCL2	*CDK4*	*EGFR*	*FGFR3*	*KIT*	*MEN1*	*MYC*	*PIK3CA*	*RET*	
BRAF	*CDK6*	*ERBB2*	*IDH1*	*KMT2C*	*MET*	*NF1*	*PIK3CB*	*ROS1*	

Table S2. Molecular profile of successfully grafted primary tumors.

CASE	TYPE	MUTATIONS
**105**	SCC	TP53:NM_000546:exon5:c.469G>T:p.V157F<br>KMT2C:NM_170606:exon7:c.918T>G:p.Y306*
**110**	SCC	TP53:NM_000546:exon5:c.413C>T:p.A138V<br>ALK:NM_004304:exon16:c.2712T>A:p.H904Q
**112**	PLEIO/AdCA	CTNNB1:NM_001098209:exon3:c.98C>T:p.S33F<br>TP53 NM_000546:exon8:c.811G>T:p.E271*<br>STK11:NM_000455:exon4:c.487G>T:p.G163C<br>KDR:NM_002253:exon20:c.2761delinsAT:p.F921Ifs*13<br>KDR:NM_002253:exon20:c.2757C>G:p.C919W
**302**	SCC	KIT:NM_000222:exon18:c.2508G>T:p.M836I<br>EGFR:NM_201282:exon10:c.1150A>T:p.T384S<br>MEN1:NM_000244:exon2:c.184A>G:p.T62A<br>MET:NM_000245:exon3:c.1241A>G:p.D414G
**508**	LCNEC	PTEN NM_000314:exon7:c.635-1G>T<br>TP53:NM_000546:exon6:c.610G>T:p.E204*<br>MET:NM_000245:exon2:c.1024C>G:p.L342V<br>CHEK1 NM_001274:exon13:c.1336-4A>G
**512**	SCC	TP53: NM_000546:exon8:c.845G>C:p.R282P<br>CDKN2A:NM_000077:exon2:c.316_317T:p.V106Cfs*39<br>PDGFRA:NM_006206:exon7:c.1102G>C:p.E368Q
**513**	SCC	TP53:NM_000546:exon5:c.469G>T:p.V157F<br>KMT2C:NM_170606:exon37:c.7201G>A:p.A2401T<br>NOTCH1:NM_017617:exon7:c.1177_1180G:p.N393del
**514**	SCC	MSH6:NM_001281492:exon2:c.1093C>T:p.R365*
**517**	SCC	RB1:NM_000321:exon8:c.852delinsTA:p.I285Nfs*2
**518**	SCC	TP53:NM_000546:exon6:c.659A>G:p.Y220C
**519**	LCNEC	RB1:NM_000321:exon19:c.1953_1954T:p.V654Cfs*3
**522**	SCC	PIK3CA:NM_006218:exon10:c.1633G>A:p.E545K<br>TP53:NM_001126115:exon1:c.92A>G:p.Y31C
**523**	AdCA	TP53:NM_000546:exon8:c.843C>G:p.D281E<br>PIK3CA:NM_006218:exon10:c.1637A>C:p.Q546P
**528**	AdCA	EGFR:NM_005228:exon19:c.2236_2238G:p.E746Vfs*15
**530**	AdCA	KRAS:NM_004985:exon3:c.183A>C:p.Q61H
**531**	SCC	TP53:NM_000546:exon7:c.747G>T:p.R249S<br>CDKN2A:NM_000077:exon2:c.262G>T:p.E88*<br>PIK3CA:NM_006218:exon10:c.1633G>A:p.E545K
**535**	AdCA	TP53 NM_000546:exon10:c.994-2A>G<br>PIK3CA:NM_006218:exon10:c.1633G>A:p.E545K
**538**	SCC	None Found
**546**	AdCA	KRAS:NM_004985:exon2:c.35G>A:p.G12D<br>ATM:NM_000051:exon62:c.8851G>A:p.V2951I
**547**	SCC	TP53 NM_000546:exon4:c.97-1G>C<br>ROS1:NM_002944:exon18:c.2704A>T:p.R902W<br>ALK:NM_004304:exon25:c.3823C>T:p.R1275*
**548**	SCC	PTEN:NM_000314:exon6:c.518G>C:p.R173P<br>IDH2:NM_002168:exon1:c.47G>A:p.G16D
**551**	SCC	MTOR:NM_004958:exon9:c.1333A>G:p.R445G

		CDKN2A:NM_058197:exon1:c.61G>C:p.A21P
**556**	AdCA	TP53:NM_000546:exon6:c.632C>T:p.T211I<br>EGFR:NM_005228:exon20:c.2300delinsCCAGCGTGGA:p.D770_N771insSVD
**559**	AdCA	KRAS:NM_004985:exon2:c.34G>T:p.G12C<br>TP53 NM_001276761:exon10:c.877-2A>T
**560**	AdCA	TP53: NM_000546:exon7:c.712T>A:p.C238S<br>STK11:NM_000455:exon1:c.180C>A:p.Y60*<br>SMAD4:NM_005359:exon11:c.1381C>T:p.Q461*
**566**	SARC	PIK3CA:NM_006218:exon5:c.1034A>C:p.N345T
**568**	AdCA	TP53:NM_000546:exon8:c.880G>T:p.E294 *<br>KRAS:NM_004985:exon2:c.34G>T:p.G12C
**569**	SCC	NF1 NM_000267:exon35:c.4662-1G>T<br>TP53 NM_000546:exon10:c.994-2A>T;<br>SMAD4:NM_005359:exon11:c.1326G>T:p.Q442H
**571**	SCC	CHEK2:NM_001349956:exon11:c.1114C>T:p.Q372*<br>TP53 NM_000546:exon10:c.994-2A>T<br>BRCA2:NM_000059:exon11:c.3668A>G:p.H1223R<br>NOTCH1:NM_017617:exon13:c.2058_2059T:p.C687Afs*84
**574**	SARC	TP53:NM_001126115:exon4:c.496G>T:p.E166* ERBB2:NM_001289937:exon22:c.2719A>T:p.S907C<br>CDKN2A:NM_000077:exon2:c.262_263A:p.E88Rfs*57<br>MET:NM_001324402:exon14:c.1865A>G:p.H622R
